# Supplementary material for: Rapid methods for identifying barriers and solutions to improve access to community health services: a scoping review protocol
Source: BMJ Open. 2023 Mar 10;13(3):e066804. doi: 10.1136/bmjopen-2022-066804 (PMC10008441; doi:10.1136/bmjopen-2022-066804)
Supplement: Supplementary data [file bmjopen-2022-066804supp001.pdf]

Appendix: Search strategies

CENTRAL on the Cochrane Library

#1	MeSH descriptor: [Health Services Accessibility] this term only
#2	MeSH descriptor: [Health Equity] this term only
#3	MeSH descriptor: [Social Determinants of Health] this term only
#4	social NEAR/2 determinant NEAR/2 health*
#5	(health* or social* or racial* or ethnic*) NEAR/5 (inequalit* or inequit* or disparit* or equit* or disadvantage* or depriv*)
#6	disadvant* or marginali* or underserved or under served or impoverish* or minorit* or racial* or ethnic*
#7	barrier*
#8	(solution* or improve* or strateg* or access* or challeng*):ti
#9	MeSH descriptor: [Community-Based Participatory Research] this term only
#10	MeSH descriptor: [Community-Institutional Relations] this term only
#11	communit* NEAR/3 (engag* or participat*)
#12	CBPR
#13	participat* NEAR/2 health NEAR/2 research
#14	communit* NEAR/2 academic NEAR/2 partnership*
#15	collective NEAR/2 empower*
#16	equity NEAR/2 mobili* NEAR/2 partnership* NEAR/2 communit*
#17	ethnograph* or communitarian*
#18	MeSH descriptor: [Interviews as Topic] this term only
#19	MeSH descriptor: [Patient Health Questionnaire] this term only
#20	MeSH descriptor: [Self Report] this term only
#21	MeSH descriptor: [Q-Sort] this term only
#22	Q-Sort
#23	Q-methodolog*
#24	system NEAR/2 dynamic NEAR/2 model*
#25	nominal NEAR/2 group* NEAR/2 technique*

#26 #1 or #2 or #3 or #4 or #5 or #6 or #7 or #8 or #9 or #10 or #11 or #12 or #13 or #14 or #15 or #16 or #17 or #18 or #19 or #20 or #21 or #22 or #23 or #24 or #25

#27 MeSH descriptor: [Problem Solving] this term only

#28 (rapid* or agile) NEAR/2 (appraisal* or assessment* or approach* or evaluation* or evaluate* or technique* or tool* or method* or research*)

#29 #27 or #28

#30 #26 and #29

#31 in vitro

#32 assay* or microb*

#33 MeSH descriptor: [Critical Care] this term only

#34 #31 or #32 or #33

#35 #30 not #34

MEDLINE Ovid

1. Health Services Accessibility/
2. Health Equity/
3. Social Determinants of Health/
4. (social adj2 determinant adj2 health$).tw.
5. ((health$ or social$ or racial$ or ethnic$) adj5 (inequalit$ or inequit$ or disparit$ or equit$ or disadvantage$ or depriv$)).tw.
6. (disadvant$ or marginali$ or underserved or under served or impoverish$ or minorit$ or racial$ or ethnic$).tw.
7. barrier$.tw.
8. (solution$ or improve$ or strateg$ or access$ or challeng$).ti.
9. Community-Based Participatory Research/
10. Community-Institutional Relations/
11. (communit$ adj3 (engag$ or participat$)).tw.
12. CBPR.tw.
13. (participat$ adj2 health adj2 research).tw.
14. (communit$ adj2 academic adj2 partnership$).tw.
15. (collective adj2 empower$).tw.

16. (equity adj2 mobili$ adj2 partnership$ adj2 communit$).tw.
17. (ethnograph$ or communitarian$).tw.
18. Interviews as Topic/
19. Patient Health Questionnaire/
20. Self Report/
21. Q-Sort/
22. Q-Sort.tw.
23. Q-methodolog$.tw.
24. (system adj2 dynamic adj2 model$).tw.
25. (nominal adj2 group$ adj2 technique$).tw.
26. or/1-25
27. Problem Solving/
28. ((rapid$ or agile) adj2 (appraisal$ or assessment$ or approach$ or evaluation$ or evaluate$ or technique$ or tool$ or method$ or research$)).tw.
29. or/27-28
30. 26 and 29
31. in vitro.tw.
32. (assay$ or microb$).tw.
33. Critical Care/
34. or/31-33
35. 30 not 34
36. limit 35 to humans
37. limit 36 to (comment or editorial or letter)
38. 36 not 37

Embase Ovid

1. health care access/
2. unmet medical need/
3. health equity/
4. "social determinants of health"/

5. (social adj2 determinant adj2 health$).tw.
6. ((health$ or social$ or racial$ or ethnic$) adj5 (inequalit$ or inequit$ or disparit$ or equit$ or disadvantage$ or depriv$)).tw.
7. (disadvant$ or marginali$ or underserved or under served or impoverish$ or minorit$ or racial$ or ethnic$).tw.
8. barrier$.tw.
9. (solution$ or improve$ or strateg$ or access$ or challeng$).ti.
10. participatory research/
11. (communit$ adj3 (engag$ or participat$)).tw.
12. CBPR.tw.
13. (participat$ adj2 health adj2 research).tw.
14. (communit$ adj2 academic adj2 partnership$).tw.
15. (collective adj2 empower$).tw.
16. (equity adj2 mobili$ adj2 partnership$ adj2 communit$).tw.
17. (ethnograph$ or communitarian$).tw.
18. interview/
19. patient health questionnaire/
20. communication barrier/
21. Q-Sort.tw.
22. Q-methodolog$.tw.
23. (system adj2 dynamic adj2 model$).tw.
24. (nominal adj2 group$ adj2 technique$).tw.
25. or/1-24
26. problem solving/
27. ((rapid$ or agile) adj2 (appraisal$ or assessment$ or approach$ or evaluation$ or evaluate$ or technique$ or tool$ or method$ or research$)).tw.
28. or/26-27
29. 25 and 28
30. in vitro.tw.
31. (assay$ or microb$).tw.
32. intensive care/
33. or/30-32

34. 29 not 33
35. limit 34 to human
36. limit 35 to (conference abstract or conference paper or editorial or letter)
37. 35 not 36

Global Health Ovid

1. health determinants/
2. empowerment/
3. (social adj2 determinant adj2 health$).tw.
4. ((health$ or social$ or racial$ or ethnic$) adj5 (inequalit$ or inequit$ or disparit$ or equit$ or disadvantage$ or depriv$)).tw.
5. (disadvant$ or marginali$ or underserved or under served or impoverish$).tw.
6. barrier$.tw.
7. (solution$ or improve$ or strateg$ or access$ or challeng$).ti.
8. (communit$ adj3 (engag$ or participat$)).tw.
9. CBPR.tw.
10. (participat$ adj2 health adj2 research).tw.
11. (communit$ adj2 based adj2 research).tw.
12. (communit$ adj2 academic adj2 partnership$).tw.
13. (collective adj2 empower$).tw.
14. (equity adj2 mobili$ adj2 partnership$ adj2 communit$).tw.
15. (ethnograph$ or communitarian$).tw.
16. interviews/
17. questionnaires/
18. Q-Sort.tw.
19. Q-methodolog$.tw.
20. (system adj2 dynamic adj2 model$).tw.
21. (nominal adj2 group$ adj2 technique$).tw.
22. or/1-21

23. Problem Solving/
24. ((rapid$ or agile) adj2 (appraisal$ or assessment$ or approach$ or evaluation$ or evaluate$ or technique$ or tool$ or method$ or research$)).tw.
25. or/23-24
26. 22 and 25
27. man.od.
28. 26 and 27
29. in vitro.tw.
30. (assay* or microb*).tw.
31. intensive care/ or intensive care units/
32. animals.od.
33. plants.od.
34. or/29-33
35. 28 not 34
36. limit 35 to (abstract only or conference or conference paper or conference proceedings or editorial)
37. 35 not 36
