# Supplementary material for: Rapid methods for identifying barriers and solutions to improve access to community health services: a scoping review protocol
Source: BMJ Open. 2023 Mar 10;13(3):e066804. doi: 10.1136/bmjopen-2022-066804 (PMC10008441; doi:10.1136/bmjopen-2022-066804)
Supplement: Author's manuscript [file bmjopen-2022-066804.draft_revisions.pdf]

BMJ Open is committed to open peer review. As part of this commitment we make the peer review history of every article we publish publicly available.

When an article is published we post the peer reviewers' comments and the authors' responses online. We also post the versions of the paper that were used during peer review. These are the versions that the peer review comments apply to.

The versions of the paper that follow are the versions that were submitted during the peer review process. They are not the versions of record or the final published versions. They should not be cited or distributed as the published version of this manuscript.

BMJ Open is an open access journal and the full, final, typeset and author-corrected version of record of the manuscript is available on our site with no access controls, subscription charges or pay-per-view fees (http://bmjopen.bmj.com).

If you have any questions on BMJ Open's open peer review process please email info.bmjopen@bmj.com

BMJ Open

Rapid methods for identifying barriers and solutions to improve access to community health services: a scoping review protocol

Journal:	*BMJ Open*
Manuscript ID	bmjopen-2022-066804
Article Type:	Protocol
Date Submitted by the Author:	21-Jul-2022
Complete List of Authors:	Allen, Luke; University of Oxford Nuffield Department of Primary Care Health Sciences<br>Jonga, Ronald; LSHTM, Population Health and Primary Care<br>Azab , Hagar ; LSHTM, Population Health and Primary Care<br>Gordon, Iris; LSHTM, LSHTM · International Centre for Eye Health (ICEH)<br>Karanja , Sarah ; LSHTM, Population Health and Primary Care<br>Evans, Jennifer; LSHTM, Faculty of Infectious and Tropical Diseases<br>Thaker , Nam ; LSHTM, Population Health and Primary Care<br>Ramke, Jacqueline; London School of Hygiene & Tropical Medicine,<br>Bastawrous, Andrew; LSHTM, LSHTM · International Centre for Eye Health (ICEH)
Keywords:	Health policy < HEALTH SERVICES ADMINISTRATION & MANAGEMENT, QUALITATIVE RESEARCH, STATISTICS & RESEARCH METHODS

SCHOLARONE™
Manuscripts

Rapid methods for identifying barriers and solutions to improve access to community health services: a scoping review protocol

Authors

1. Luke N Allen, LSHTM, [drlukeallen@gmail.com] -Corresponding author:
   University of Oxford Nuffield Department of Primary Care Health Sciences
   Oxford, Oxfordshire, UK
   01865 289300
2. Ronald Jonga, LSHTM [ronald.jonga@mail.com]
   LSHTM, Population Health and Primary Care
   Keppel St, London WC1E 7HT
   London, London, UK
   020 7636 8636
3. Hagar Azab [hagarazab29@gmail.com]
   LSHTM, Population Health and Primary Care
   Keppel St, London WC1E 7HT
   London, London, UK
   020 7636 8636
4. Iris Gordon [Iris.Gordon@lshtm.ac.uk]
   LSHTM, Population Health and Primary Care
   Keppel St, London WC1E 7HT
   London, London, UK
   020 7636 8636
5. Sarah Karanja [sashkaranja@gmail.com]
   LSHTM, Population Health and Primary Care
   Keppel St, London WC1E 7HT
   London, London, UK
   020 7636 8636
6. Jennifer Evans [Jennifer.Evans@lshtm.ac.uk]
   LSHTM, Population Health and Primary Care
   Keppel St, London WC1E 7HT
   London, London, UK
   020 7636 8636
7. Nam Thaker [nam@peekvision.org]
   LSHTM, Population Health and Primary Care
   Keppel St, London WC1E 7HT
   London, London, UK
   020 7636 8636
8. Jacqueline Ramke [Jacqueline.ramke@lshtm.ac.uk, LSHTM and School of Optometry and Vision Science, University of Auckland, Auckland, New Zealand
   LSHTM, Population Health and Primary Care

For peer review only

Keppel St, London WC1E 7HT

London, London, UK

020 7636 8636

9. Andrew Bastawrous [Andrew.bastawrous@lshtm.ac.uk]

LSHTM, Population Health and Primary Care

Keppel St, London WC1E 7HT

London, London, UK

020 7636 8636

For peer review only

Abstract

Introduction: Low attendance rates for community health services reflect important barriers that prevent people from receiving the care they need. Services and health systems that seek to advance Universal Health Coverage need to understand and act on these factors. Formal qualitative research is the best way to elicit barriers and identify potential solutions, however traditional approaches take months to complete and can be very expensive. We aim to map the methods that have been used to rapidly elicit barriers to accessing community health services and identify potential solutions.

Methods and analysis We will search MEDLINE, Embase, the Cochrane Library, and Global Health for empirical studies that use rapid methods (<14 days) to elicit barriers and potential solutions from non-attenders. We will exclude hospital-based and 100% remotely delivered services. We will include studies conducted in any country from 1978 to present. We will not limit by language. Two reviewers will independently perform screening and data extraction, with disagreements resolved by a third reviewer. We will tabulate the different approaches used and present data on time, skills, and financial requirements for each approach, as well as the governance framework and any strengths and weaknesses presented by the study authors. We will follow JBI scoping review guidance and the PRISMA Extension for Scoping Reviews.

Ethics and dissemination: Ethical approval is not required. We will share our findings in the peer-reviewed literature, at conferences, and with WHO policymakers working in this space.

Registration: Open Science Framework (https://osf.io/a6r2m)

For peer review only

Strengths and Limitations

- As far as we are aware, this will be the first review to evaluate the rapid approaches used to elicit barriers to access community health services and potential solutions.
- Improving access and grounding service improvements in community engagement are two major global health priorities.
- Our review will follow best-practice guidelines, use a search strategy devised by an information specialist, and use independent dual review at every stage.
- We will miss rapid approaches that have been used effectively but not written about.

For peer review only

Introduction

Rationale

Many health programmes experience large mismatches between people identified with a clinical need and those who attend services. A recent international systematic review of non-attendance across all medical specialities estimated that 23% of clinic appointments are missed, with the highest rate observed in Africa (43%).[1] Low attendance rates often reflect significant barriers faced by users.[2] Marginalised populations are often the least likely to receive care.[3,4] Improving access to ensure that all individuals and communities receive the care they need lies at the heart of Universal Health Coverage - a core element in the Sustainable Development Agenda.[5,6]

When it comes to identifying barriers and potential solutions, WHO has noted that "it is the experts who identify the problems and formulate interventions, while the problems and solutions as perceived by those at particular risk rarely constitute the base for action."[7] Efforts to improve attendance rates should be grounded in an understanding of both supply- and demand-side barriers, elicited through engagement with affected communities.[2,8,9] The WHO Primary Health Care (PHC) Operational Framework defines engagement as "the process of involving people and communities in the design, planning and delivery of health services, thereby enabling them to make choices about care and treatment options or to participate in strategic decision-making on how health resources should be spent." Turk and colleagues note that health service interventions "must be done with, and not simply done to, the people affected."[10]

Research evidence aligns with common sense in finding that involving communities in the development of services improves health outcomes and sustainability.[10] For-profit enterprises seem to understand the value of engaging with their customers: many companies use focus groups and market research to continually hone their products and services to meet the evolving needs of their customer base. Our sense is that health programmes are less active in this space. Ideally - given the scale of the problem - health system managers would be able to deploy affordable, rapid, and methodologically sound tools to engage with the groups that face the highest barriers to accessing care in order to elicit their ideas for service improvements. In reality, existing qualitative elicitation and co-production techniques commonly take more than a year to plan, execute, analyse, and report. They require formal ethical review, formally trained qualitative researchers, the use of specialist software, and qualitative expertise to interpret and apply the findings. These resource requirements are prohibitive for most health system managers, and in many low-income settings there is not a ready supply of specialist expertise. This can lead to well-conducted but one-off engagement activities where the findings are inappropriately generalised to other groups. At the other end of the spectrum are tokenistic and/or methodologically flawed efforts to gather and act upon service user feedback, and multitudes of poorly designed patient satisfaction surveys. We are interested in exploring whether it is possible to obtain meaningful and robust findings with rapid tools[11]; here defined as approaches that take days rather than months to contact and recruit participants, elicit barriers, and develop a list of potential interventions to improve the service[12]. Such tools would have very wide application across a broad range of settings and would support the development of Primary Health Care (PHC) oriented systems that are built on community engagement.[13]

For peer review only

Aim and objectives

We will perform a scoping review[14] of the literature to identify, categorise, and evaluate the methods that are being used to rapidly elicit barriers and potential solutions from service users in any community-based health service. We want to understand the strengths and weaknesses of the different methods that have been used, their resource requirements, and their governance frameworks as described by their users.

Responding to the need for rapid, affordable, and scientifically robust approaches that can be used to continually improve health services, we ultimately aim to identify the minimum viable product in this space. We want to identify approaches that provide sound, non-tokenistic, and actionable intelligence with minimal time, money, equipment, personnel, and skill requirements.

Review question

What rapid methods have been used to engage with community-based health service users to elicit barriers to access and potential solutions? For each method, what are the main outputs, methodological strengths and limitations, and resource requirements in terms of time, personnel, and other costs?

For the purposes of this review, ‘community-based care’ will be defined as non-hospital inpatient care that involves interaction with a clinician, and a ‘community’ will be defined as a group who share geographies, interests, or identities. This definition is based on that used in the WHO Operational Framework for PHC and the work of Mason et al.[15] We will use the WHO Operational Framework definition for ‘community engagement’ that is presented in the introduction.[15] We note that Primary Health Care is not the same as community-based care: the former is a whole-of-society approach to health (that includes hospitals even though it focuses on primary care).[13]

Methods and analysis

Guidelines

We developed our protocol in line with the PRISMA checklist extension for scoping reviews (PRISMA-ScR)[16] and our review will be conducted in accordance with the JBI methodology, based on the principles of Arksey and O’Malley and Levac and colleagues.[17–19] Scoping reviews are the most appropriate method for mapping and characterising the available evidence in a given area, and follow five steps:[20–22]

1) Defining the research question/s

2) Identifying relevant studies

3) Study selection

4) Charting the data

5) Collating, summarising, and reporting the results

An iterative approach will be taken toward searching the literature, refining the search strategy, reviewing articles for inclusion, and extracting relevant data.[16,23–25]

For peer review only

Participants

All service users aged five years and older of outpatient and community-based services in any country, serving any need. We will include methods where engagement activities target service user lay representatives such as patient advocacy groups, parents, or village elders, however these findings will be reported separately in the findings. We will exclude methods that engage service providers, managers, or policymakers. We will include approaches that engage a mix of users and providers as long as it is possible to disaggregate the findings pertaining to service user engagement.

As we are focusing on groups that face barriers to access, we will exclude approaches that exclusively engage with people who are present at their services i.e. our focus is on methods for contacting and engaging with non-attenders.

Patient and Public Involvement

No patient and public involvement.

Concept

We are interested in methods used for engaging service users to elicit their perceptions of barriers to accessing care and generating ideas for service modifications that could improve access rates and outcomes amongst people with similar characteristics.

We are focusing on rapid methods, defined as those that can be used to deliver a list of barriers and potential solutions within 14 days or less. This is an arbitrary threshold but draws from our clinical experience in leading health services and represents what we feel to be an acceptable amount of time to generate data to inform real-time decision-making.

We will include all forms of established or novel methods from any scientific field of enquiry. We expect to find examples of the following types of method:

- Interviews: face-to-face, telephone, videocall
- Focus groups
- Group system dynamic modelling
- Q methodology
- Nominal group technique
- Surveys: in-person, web, telephone, text message
- Rapid ethnography

Context

We are not limiting the review to any specific population, culture or geography. We will include studies from all countries and any setting except hospital inpatients. We will exclude services that are delivered remotely, compulsory care such as when patients are sectioned for mental health care, and services where no interaction with a clinician is required, such as automated services to obtain self-testing kits.

For peer review only

Types of Sources

We will include all empirical study types that report on the use of a given method to elicit barriers or potential solutions with a maximum of 14 days between commencing fieldwork and generating the findings.

We will exclude methodological texts, reviews, letters, and conference abstracts. We will also exclude systematic reviews, but we will search their reference lists and include any underlying primary studies that meet our inclusion criteria.

Search strategy

The search strategy will be built around rapid community-based methods and access to health services [2627](Box 1). The search will be limited to human studies published since 1978; the year of the Alma-Ata Declaration on Primary Health Care. We will search for full-text studies published in any language. We plan to complete the review by December 2022. The search strategy results will be presented in a PRISMA flowchart that will show how studies were eliminated until final search yield that will constitute the basis for synthesis.

We will search the following information resources: the Cochrane Library, MEDLINE Ovid, Embase Ovid and Global Health Ovid. The first 20 pages of Google Scholar will also be screened. The search strategy, including all identified keywords and index terms, will be adapted for each included database and/or information source. Box 1 presents the search strategy for Medline. The Appendix presents the tailored search strategies for all databases. We will check the reference lists of included studies and relevant systematic reviews to identify any additional potentially relevant reports of studies. Key authors will be contacted to uncover additional or upcoming studies.

Box 1: Search terms used for Medline

1. Health Services Accessibility/
2. Health Equity/
3. Social Determinants of Health/
4. (social adj2 determinant adj2 health$).tw.
5. ((health$ or social$ or racial$ or ethnic$) adj5 (inequalit$ or inequit$ or disparit$ or equit$ or disadvantage$ or depriv$)).tw.
6. (disadvant$ or marginali$ or underserved or under served or impoverish$ or minorit$ or racial$ or ethnic$).tw.
7. barrier$.tw.
8. (solution$ or improve$ or strateg$ or access$ or challeng$).ti.
9. Community-Based Participatory Research/
10. Community-Institutional Relations/
11. (communit$ adj3 (engag$ or participat$)).tw.
12. CBPR.tw.
13. (participat$ adj2 health adj2 research).tw.
14. (communit$ adj2 academic adj2 partnership$).tw.
15. (collective adj2 empower$).tw.
16. (equity adj2 mobili$ adj2 partnership$ adj2 communit$).tw.
17. (ethnograph$ or communitarian$).tw.
18. Interviews as Topic/

For peer review only - http://bmjopen.bmj.com/site/about/guidelines.xhtml

19. *Patient Health Questionnaire/*
20. *Self Report/*
21. *Q-Sort/*
22. *Q-Sort.tw.*
23. *Q-methodolog$.tw.*
24. *(system adj2 dynamic adj2 model$).tw.*
25. *(nominal adj2 group$ adj2 technique$).tw.*
26. *or/1-25*
27. *Problem Solving/*
28. *((rapid$ or agile) adj2 (appraisal$ or assessment$ or approach$ or evaluation$ or evaluate$ or technique$ or tool$ or method$ or research$)).tw.*
29. *or/27-28*
30. *26 and 29*
31. *in vitro.tw.*
32. *(assay$ or microb$).tw.*
33. *Critical Care/*
34. *or/31-33*
35. *30 not 34*
36. *limit 35 to humans*
37. *limit 36 to (comment or editorial or letter)*
38. *36 not 37*
39. *limit 38 to yr="1978 -Current"*

Study/Source of Evidence selection

Following the search, all identified citations will be collated and uploaded into Covidence (Veritas Health Innovation, Melbourne) and duplicates will be removed. Following a pilot test, titles and abstracts will then be screened by two independent reviewers for assessment against the inclusion criteria. Studies that clearly do not meet the inclusion criteria will be excluded. The reviewers will meet after every 10% batch of papers has been screened to discuss any issues. Any disagreements will be resolved through consensus-based discussion, or if necessary, discussion with a third reviewer.

We will obtain full texts for the potentially relevant papers. Two review authors will independently assess the papers against the inclusion criteria to determine their eligibility for inclusion. Non-English language papers will be translated into English using Google translate. The review authors will resolve disagreements through consensus-based discussion, or if necessary, discussion with a third reviewer. The reviewers will record reasons for exclusion at the full-text screening stage.

The results of the search and the study inclusion process will be reported in full in the final scoping review and presented in a PRISMA flow diagram.[28]

Data Extraction

Two review authors will independently extract study characteristics and data from the included studies using a data extraction form developed by the reviewers. The data extraction form will be piloted on three studies by two review authors and required amendments will be made by consensus [29]. We anticipate a broad scope of included studies, so data charting will be an iterative process throughout the review. The data extraction tool will be modified and revised as necessary during the process of extracting data from each included evidence source. Any discrepancies will be

resolved by group discussion. Modifications will be detailed in the scoping review. Where required, authors of papers will be contacted to request missing or additional data.

The data extracted will include specific details about the participants, concept, context, study methods and key findings relevant to the review question:

- Article title
- Journal title
- Authors
- Country
- Language
- Publication year
- Study type
- Type of approach (e.g. focus group) and description.
  - setting,
  - participants,
  - facilitators
- Main output if anything other than a prioritised list of potential service modifications
- Methodological strengths and limitations, as documented by the authors
- Resource requirements:
  - Number of personnel, and essential skills/level of training
  - Number of days for each person, full time equivalent
  - Total number of days taken from conception to findings; including planning, recruitment, engagement, and analysis stages
  - Equipment
  - Total financial cost
- Framework used to structure interaction and elicit barriers and solutions
- Method of recording (notes, audio etc)
- Other practical requirements or qualitative considerations reported in-text
- Ethics and governance requirements
- Level, form, frequency, and intensity of participation:
  - Level of participation will be assessed using the five categories used by WHO: inform, consult, involve, collaborate, and empower (Box 1).
  - Form will be assessed using the four categories used by WHO: community-oriented, -based, -managed, and –owned.
  - Frequency is defined as the number of discrete interactions between the project team and the service users.
  - Intensity represents the extent to which participants interact, exchange information and influence decision-making in participation processes [30].
- Power relations, prevailing knowledge, and beliefs and cultural barriers[10], described by the authors.
- Any documented power relations, prevailing knowledge, and beliefs and cultural barriers.

Data Analysis and Presentation

We plan to conduct a formal narrative descriptive synthesis without meta-analysis. We will stratify the synthesis by methodological approach. We will present a summary table of the different

For peer review only - http://bmjopen.bmj.com/site/about/guidelines.xhtml

methods used, grouped by discipline. We will also tabulate the resource requirements, form of participation, and methodological strengths and limitations. Quantitative resource requirement data will be presented in whole numbers, days and 2022 US dollar amounts as appropriate. Ratios will be used to compare costs between approaches. Qualitative outcomes will be presented narratively. Methods used to engage with service users and service user representatives will be presented separately.

We will not conduct methodological quality assessment of included studies, in keeping with usual practice for scoping reviews.[14,17]

Ethics and Dissemination

Ethical approval is not required. We will share our findings in the peer-reviewed literature, at conferences, and with WHO policymakers working in this space.

Contributions

LNA conceptualised and planned the study with SK, IG, JE, NT and AB. IG and LNA designed the search terms with input from RJ, HA, SK, JE, JR and NT. LNA wrote the first draft with JR. HA, IG, SK, JE, NT, JR and AB critically revised iterations of the manuscript. All authors read and approved the final protocol.

Funding

This work was supported by the National Institute for Health Research (NIHR) (using the UK's Official Development Assistance (ODA) Funding) and Wellcome [grant reference 215633/Z/19/Z] under the NIHR-Wellcome Partnership for Global Health Research. The views expressed are those of the authors and not necessarily those of Wellcome, the NIHR or the Department of Health and Social Care. The study was sponsored by the London School of Hygiene & Tropical Medicine. The funders and study sponsor had no role in developing the protocol.

Competing interests

There are no competing interests to report for any of the authors.

References

For peer review only

1. Dantas LF, Fleck JL, Cyrino Oliveira FL, Hamacher S. No-shows in appointment scheduling - a systematic literature review. Health Policy Amst Neth. 2018 Apr;122(4):412–21.

2. Levesque JF, Harris MF, Russell G. Patient-centred access to health care: conceptualising access at the interface of health systems and populations. Int J Equity Health. 2013 Mar 11;12(1):18.

3. Hart JT. The Inverse care law. The Lancet. 1971 Feb 27;297(7696):405–12.

4. World Health Organization. Closing the gap in a generation: health equity through action on the social determinants of health - Final report of the commission on social determinants of health [Internet]. Geneva; 2008 [cited 2021 Nov 11]. Available from: https://www.who.int/publications-detail-redirect/WHO-IER-CSDH-08.1

5. World Health Organization. Universal health coverage (UHC) [Internet]. 2021 [cited 2021 Nov 11]. Available from: https://www.who.int/news-room/fact-sheets/detail/universal-health-coverage-(uhc)

6. UN General Assembly. A/RES/70/1: Transforming our world: the 2030 Agenda for Sustainable Development [Internet]. 2015 Sep [cited 2021 Nov 11]. Available from: https://www.un.org/ga/search/view_doc.asp?symbol=A/RES/70/1&Lang=E

7. WHO Europe. Toolkit on social participation. [Internet]. 2016 [cited 2022 Apr 8]. Available from: https://www.euro.who.int/en/publications/abstracts/toolkit-on-social-participation.-methods-and-techniques-for-ensuring-the-social-participation-of-roma-populations-and-other-social-groups-in-the-design,-implementation,-monitoring-and-evaluation-of-policies-and-programmes-to-improve-their-health-2016

8. WHO and UNICEF. Declaration of Alma-Ata [Internet]. 1978 [cited 2022 Mar 23]. Available from: https://www.who.int/teams/social-determinants-of-health/declaration-of-alma-ata

9. WHO and UNICEF. Declaration of Astana on Primary Health Care [Internet]. 2018 [cited 2022 Mar 23]. Available from: https://www.who.int/teams/primary-health-care/conference/declaration

10. Turk E, Durrance-Bagale A, Han E, Bell S, Rajan S, Lota MMM, et al. International experiences with co-production and people centredness offer lessons for covid-19 responses. BMJ. 2021 Feb 15;372:m4752.

11. Miles McNall, Pennie G Foster-Fishmanhttps://journals.sagepub.com/doi/abs/10.1177/1098214007300895?journalCode=ajec https://journals.sagepub.com/doi/abs/10.1177/1098214007300895?journalCode=ajec. Methods of Rapid Evaluation, Assessment, and Appraisal. Available from: https://journals.sagepub.com/doi/abs/10.1177/1098214007300895?journalCode=ajec

12. George AS, Mehra V, Scott K, Sriram V. Community Participation in Health Systems Research: A Systematic Review Assessing the State of Research, the Nature of Interventions Involved and the Features of Engagement with Communities. PloS One. 2015;10(10):e0141091.

13. WHO. A vision for primary health care in the 21st century: towards universal health coverage and the Sustainable Development Goals [Internet]. 2018 [cited 2022 Mar 23]. Available from: https://apps.who.int/iris/handle/10665/328065

For peer review only

14. Song E, Ang L, Park JY, Jun EY, Kim KH, Jun J, et al. A scoping review on biomedical journal peer review guides for reviewers. PloS One. 2021;16(5):e0251440.

15. WHO. Operational Framework for Primary Health Care Transforming Vision Into Action [Internet]. 2008. Available from: file:///C:/Users/rjonga/Downloads/9789240017832-eng.pdf

16. Tricco AC, Lillie E, Zarin W, O'Brien KK, Colquhoun H, Levac D, et al. PRISMA Extension for Scoping Reviews (PRISMA-ScR): Checklist and Explanation. Ann Intern Med. 2018 Oct 2;169(7):467–73.

17. Peters M, Godfrey C, McInerney P. The Joanna Briggs Institute Reviewers' Manual 2015 Methodology for JBI Scoping Reviews [Internet]. Available from: https://nursing.lsuhsc.edu/JBI/docs/ReviewersManuals/Scoping-.pdf

18. Arksey H, O'Malley L. Scoping studies: towards a methodological framework. Int J Soc Res Methodol. 2005 Feb 1;8(1):19–32.

19. Levac D, Colquhoun H, O'Brien KK. Scoping studies: advancing the methodology. Implement Sci. 2010 Dec;5(1):1–9.

20. Khalil H, Peters M, Godfrey CM, McInerney P, Soares CB, Parker D. An Evidence-Based Approach to Scoping Reviews. Worldviews Evid Based Nurs. 2016 Apr;13(2):118–23.

21. Munn Z, Peters MDJ, Stern C, Tufanaru C, McArthur A, Aromataris E. Systematic review or scoping review? Guidance for authors when choosing between a systematic or scoping review approach. BMC Med Res Methodol. 2018 Nov 19;18(1):143.

22. Peterson J, Pearce PF, Ferguson LA, Langford CA. Understanding scoping reviews: Definition, purpose, and process. J Am Assoc Nurse Pract. 2017 Jan;29(1):12–6.

23. Cooper C, Booth A, Varley-Campbell J, Britten N, Garside R. Defining the process to literature searching in systematic reviews: a literature review of guidance and supporting studies. BMC Med Res Methodol. 2018 Aug 14;18(1):85.

24. Garcia-Perdomo HA. Information sources and search strategy pitfalls to avoid in systematic reviews. Eur J Cancer Prev Off J Eur Cancer Prev Organ ECP. 2021 Nov 1;30(6):481.

25. Mueller M, D'Addario M, Egger M, Cevallos M, Dekkers O, Mugglin C, et al. Methods to systematically review and meta-analyse observational studies: a systematic scoping review of recommendations. BMC Med Res Methodol. 2018 May 21;18(1):44.

26. Said M, Boardman G, Kidd S. Barriers to accessing mental health services in Somali-Australian women: a qualitative study. Int J Ment Health Nurs. 2021 Aug;30(4):931–8.

27. Memon A, Taylor K, Mohebati LM, Sundin J, Cooper M, Scanlon T, et al. Perceived barriers to accessing mental health services among black and minority ethnic (BME) communities: a qualitative study in Southeast England. BMJ Open. 2016 Nov 16;6(11):e012337.

28. Page MJ, McKenzie JE, Bossuyt PM, Boutron I, Hoffmann TC, Mulrow CD, et al. The PRISMA 2020 statement: an updated guideline for reporting systematic reviews. BMJ. 2021 Mar 29;372:n71.

For peer review only

29. Cochrane Collaboration. Data Collection Form [Internet]. Available from: https://training.cochrane.org/sites/training.cochrane.org/files/public/uploads/resources/downloadable_resources/English/Collecting%20data%20-%20form%20for%20RCTs%20only.doc

30. World Health Organisation. TOOLKIT ON SOCIAL PARTICIPATION [Internet]. Available from: https://www.euro.who.int/__data/assets/pdf_file/0003/307452/Toolkit-social-partecipation.pdf

For peer review only

For peer review only

Appendix: Search strategies

CENTRAL on the Cochrane Library

#1 MeSH descriptor: [Health Services Accessibility] this term only

#2 MeSH descriptor: [Health Equity] this term only

#3 MeSH descriptor: [Social Determinants of Health] this term only

#4 social NEAR/2 determinant NEAR/2 health*

#5 (health* or social* or racial* or ethnic*) NEAR/5 (inequalit* or inequit* or disparit* or equit* or disadvantage* or depriv*)

#6 disadvant* or marginali* or underserved or under served or impoverish* or minorit* or racial* or ethnic*

#7 barrier*

#8 (solution* or improve* or strateg* or access* or challeng*):ti

#9 MeSH descriptor: [Community-Based Participatory Research] this term only

#10 MeSH descriptor: [Community-Institutional Relations] this term only

#11 communit* NEAR/3 (engag* or participat*)

#12 CBPR

#13 participat* NEAR/2 health NEAR/2 research

#14 communit* NEAR/2 academic NEAR/2 partnership*

#15 collective NEAR/2 empower*

#16 equity NEAR/2 mobili* NEAR/2 partnership* NEAR/2 communit*

#17 ethnograph* or communitarian*

#18 MeSH descriptor: [Interviews as Topic] this term only

#19 MeSH descriptor: [Patient Health Questionnaire] this term only

#20 MeSH descriptor: [Self Report] this term only

#21 MeSH descriptor: [Q-Sort] this term only

#22 Q-Sort

#23 Q-methodolog*

#24 system NEAR/2 dynamic NEAR/2 model*

#25 nominal NEAR/2 group* NEAR/2 technique*

For peer review only

#26 #1 or #2 or #3 or #4 or #5 or #6 or #7 or #8 or #9 or #10 or #11 or #12 or #13 or #14 or #15 or #16 or #17 or #18 or #19 or #20 or #21 or #22 or #23 or #24 or #25

#27 MeSH descriptor: [Problem Solving] this term only

#28 (rapid* or agile) NEAR/2 (appraisal* or assessment* or approach* or evaluation* or evaluate* or technique* or tool* or method* or research*)

#29 #27 or #28

#30 #26 and #29

#31 in vitro

#32 assay* or microb*

#33 MeSH descriptor: [Critical Care] this term only

#34 #31 or #32 or #33

#35 #30 not #34

MEDLINE Ovid

1. Health Services Accessibility/
2. Health Equity/
3. Social Determinants of Health/
4. (social adj2 determinant adj2 health$).tw.
5. ((health$ or social$ or racial$ or ethnic$) adj5 (inequalit$ or inequit$ or disparit$ or equit$ or disadvantage$ or depriv$)).tw.
6. (disadvant$ or marginali$ or underserved or under served or impoverish$ or minorit$ or racial$ or ethnic$).tw.
7. barrier$.tw.
8. (solution$ or improve$ or strateg$ or access$ or challeng$).ti.
9. Community-Based Participatory Research/
10. Community-Institutional Relations/
11. (communit$ adj3 (engag$ or participat$)).tw.
12. CBPR.tw.
13. (participat$ adj2 health adj2 research).tw.
14. (communit$ adj2 academic adj2 partnership$).tw.
15. (collective adj2 empower$).tw.

For peer review only

16. (equity adj2 mobili$ adj2 partnership$ adj2 communit$).tw.
17. (ethnograph$ or communitarian$).tw.
18. Interviews as Topic/
19. Patient Health Questionnaire/
20. Self Report/
21. Q-Sort/
22. Q-Sort.tw.
23. Q-methodolog$.tw.
24. (system adj2 dynamic adj2 model$).tw.
25. (nominal adj2 group$ adj2 technique$).tw.
26. or/1-25
27. Problem Solving/
28. ((rapid$ or agile) adj2 (appraisal$ or assessment$ or approach$ or evaluation$ or evaluate$ or technique$ or tool$ or method$ or research$)).tw.
29. or/27-28
30. 26 and 29
31. in vitro.tw.
32. (assay$ or microb$).tw.
33. Critical Care/
34. or/31-33
35. 30 not 34
36. limit 35 to humans
37. limit 36 to (comment or editorial or letter)
38. 36 not 37

Embase Ovid

1. health care access/
2. unmet medical need/
3. health equity/
4. "social determinants of health"/

For peer review only

5. (social adj2 determinant adj2 health$).tw.

6. ((health$ or social$ or racial$ or ethnic$) adj5 (inequalit$ or inequit$ or disparit$ or equit$ or disadvantage$ or depriv$)).tw.

7. (disadvant$ or marginali$ or underserved or under served or impoverish$ or minorit$ or racial$ or ethnic$).tw.

8. barrier$.tw.

9. (solution$ or improve$ or strateg$ or access$ or challeng$).ti.

10. participatory research/

11. (communit$ adj3 (engag$ or participat$)).tw.

12. CBPR.tw.

13. (participat$ adj2 health adj2 research).tw.

14. (communit$ adj2 academic adj2 partnership$).tw.

15. (collective adj2 empower$).tw.

16. (equity adj2 mobili$ adj2 partnership$ adj2 communit$).tw.

17. (ethnograph$ or communitarian$).tw.

18. interview/

19. patient health questionnaire/

20. communication barrier/

21. Q-Sort.tw.

22. Q-methodolog$.tw.

23. (system adj2 dynamic adj2 model$).tw.

24. (nominal adj2 group$ adj2 technique$).tw.

25. or/1-24

26. problem solving/

27. ((rapid$ or agile) adj2 (appraisal$ or assessment$ or approach$ or evaluation$ or evaluate$ or technique$ or tool$ or method$ or research$)).tw.

28. or/26-27

29. 25 and 28

30. in vitro.tw.

31. (assay$ or microb$).tw.

32. intensive care/

33. or/30-32

For peer review only

34. 29 not 33
35. limit 34 to human
36. limit 35 to (conference abstract or conference paper or editorial or letter)
37. 35 not 36

Global Health Ovid

1. health determinants/
2. empowerment/
3. (social adj2 determinant adj2 health$).tw.
4. ((health$ or social$ or racial$ or ethnic$) adj5 (inequalit$ or inequit$ or disparit$ or equit$ or disadvantage$ or depriv$)).tw.
5. (disadvant$ or marginali$ or underserved or under served or impoverish$).tw.
6. barrier$.tw.
7. (solution$ or improve$ or strateg$ or access$ or challeng$).ti.
8. (communit$ adj3 (engag$ or participat$)).tw.
9. CBPR.tw.
10. (participat$ adj2 health adj2 research).tw.
11. (communit$ adj2 based adj2 research).tw.
12. (communit$ adj2 academic adj2 partnership$).tw.
13. (collective adj2 empower$).tw.
14. (equity adj2 mobili$ adj2 partnership$ adj2 communit$).tw.
15. (ethnograph$ or communitarian$).tw.
16. interviews/
17. questionnaires/
18. Q-Sort.tw.
19. Q-methodolog$.tw.
20. (system adj2 dynamic adj2 model$).tw.
21. (nominal adj2 group$ adj2 technique$).tw.
22. or/1-21

For peer review only

23. Problem Solving/

24. ((rapid$ or agile) adj2 (appraisal$ or assessment$ or approach$ or evaluation$ or evaluate$ or technique$ or tool$ or method$ or research$)).tw.

25. or/23-24

26. 22 and 25

27. man.od.

28. 26 and 27

29. in vitro.tw.

30. (assay* or microb*).tw.

31. intensive care/ or intensive care units/

32. animals.od.

33. plants.od.

34. or/29-33

35. 28 not 34

36. limit 35 to (abstract only or conference or conference paper or conference proceedings or editorial)

37. 35 not 36

For peer review only

BMJ Open

Rapid methods for identifying barriers and solutions to improve access to community health services: a scoping review protocol

Journal:	*BMJ Open*
Manuscript ID	bmjopen-2022-066804.R1
Article Type:	Protocol
Date Submitted by the Author:	05-Feb-2023

Complete List of Authors:	Allen, Luke; University of Oxford Nuffield Department of Primary Care Health Sciences<br>Azab , Hagar ; LSHTM, Population Health and Primary Care<br>Jonga, Ronald; LSHTM, Population Health and Primary Care<br>Gordon, Iris; LSHTM, LSHTM · International Centre for Eye Health (ICEH)<br>Karanja , Sarah ; LSHTM, Population Health and Primary Care<br>Evans, Jennifer; LSHTM, Faculty of Infectious and Tropical Diseases<br>Thaker , Nam ; LSHTM, Population Health and Primary Care<br>Ramke, Jacqueline; London School of Hygiene & Tropical Medicine,<br>Bastawrous, Andrew; LSHTM, LSHTM · International Centre for Eye Health (ICEH)

**Primary Subject Heading**:	Global health
Secondary Subject Heading:	Global health, Health services research, Qualitative research
Keywords:	Health policy < HEALTH SERVICES ADMINISTRATION & MANAGEMENT, STATISTICS & RESEARCH METHODS, Health Services Accessibility, QUALITATIVE RESEARCH

SCHOLARONE™
Manuscripts

Rapid methods for identifying barriers and solutions to improve access to community health services: a scoping review protocol

Authors

1. Luke N Allen, LSHTM, [drlukeallen@gmail.com], corresponding author
    a. LSHTM, Keppel Street, London, WC1E 7HT, United Kingdom
    b. Phone: +44 (0)20 7636 8636
    c. Fax: +44 (0)20 7436 5389
2. Ronald Jonga, LSHTM [ronald.jonga@mail.com]
    a. LSHTM, Keppel Street, London, WC1E 7HT, United Kingdom]
3. Hagar Azab [hagarazab29@gmail.com]
    a. WHO EMRO, Cairo, Egypt
4. Iris Gordon [Iris.Gordon@lshtm.ac.uk]
    a. LSHTM, Keppel Street, London, WC1E 7HT, United Kingdom]
5. Sarah Karanja [sashkaranja@gmail.com]
    a. KEMRI, Kenya
6. Jennifer Evans [Jennifer.Evans@lshtm.ac.uk]
    a. LSHTM, Keppel Street, London, WC1E 7HT, United Kingdom]
7. Nam Thaker [nam@peekvision.org]
    a. LSHTM, Keppel Street, London, WC1E 7HT, United Kingdom]
8. Jacqueline Ramke [Jacqueline.ramke@lshtm.ac.uk]
    a. LSHTM, Keppel Street, London, WC1E 7HT, United Kingdom]
9. Andrew Bastawrous [Andrew.bastawrous@lshtm.ac.uk]
    a. LSHTM, Keppel Street, London, WC1E 7HT, United Kingdom]

Keywords

Health Services Accessibility, Health Equity, Social Determinants of Health, Barriers to Access, Community Care

Word count

1. 1

For peer review only

2910

1. 2

For peer review only

For peer review only - http://bmjopen.bmj.com/site/about/guidelines.xhtml

Abstract

Objectives: Low attendance rates for community health services reflect important barriers that prevent people from receiving the care they need. Services and health systems that seek to advance Universal Health Coverage need to understand and act on these factors. Formal qualitative research is the best way to elicit barriers and identify potential solutions, however traditional approaches take months to complete and can be very expensive. We aim to map the methods that have been used to rapidly elicit barriers to accessing community health services and identify potential solutions.

Methods and analysis We will search MEDLINE, Embase, the Cochrane Library, and Global Health for empirical studies that use rapid methods (<14 days) to elicit barriers and potential solutions from intended service beneficiaries. We will exclude hospital-based and 100% remotely delivered services. We will include studies conducted in any country from 1978 to present. We will not limit by language. Two reviewers will independently perform screening and data extraction, with disagreements resolved by a third reviewer. We will tabulate the different approaches used and present data on time, skills, and financial requirements for each approach, as well as the governance framework and any strengths and weaknesses presented by the study authors. We will follow JBI scoping review guidance and report the review using the PRISMA Extension for Scoping Reviews.

Ethics and dissemination: Ethical approval is not required. We will share our findings in the peer-reviewed literature, at conferences, and with WHO policymakers working in this space.

Registration: Open Science Framework (https://osf.io/a6r2m)

For peer review only

Strengths and Limitations

- As far as we are aware, this will be the first review to evaluate the rapid approaches used to elicit barriers to access community health services and potential solutions.
- Improving access and grounding service improvements in community engagement are two major global health priorities.
- Our review will follow best-practice guidelines, use a search strategy devised by an information specialist, and use independent dual review at every stage.
- We will miss rapid approaches that have been used effectively but not written about, and those that take longer than 14 days to deliver findings.

For peer review only

For peer review only - http://bmjopen.bmj.com/site/about/guidelines.xhtml

Introduction

Rationale

Many health programmes experience large mismatches between people identified with a clinical need and those who attend services. A recent international systematic review of non-attendance across all medical specialities estimated that 23% of clinic appointments are missed, with the highest rate observed in Africa (43%).[1] Low attendance rates often reflect significant barriers faced by users.[2] Marginalised populations are often the least likely to receive care.[3,4] Improving access to ensure that all individuals and communities receive the care they need lies at the heart of Universal Health Coverage - a core element in the Sustainable Development Agenda.[5,6]

Complex supply and demand factors govern access to health services and multiple frameworks have been developed, typically defining access as the ability to perceive, seek, reach, pay for and engage with care.[2,7–11] Access is increasingly being extended through the use of digital services and remote consultations.[12,13] Whilst these services are useful, they come with their own set of barriers and equity issues, and cannot fully replace the central role played by in-person clinical providers.[12,14] When it comes to identifying barriers to attending in-person clinical services and potential solutions, WHO has noted that "it is the experts who identify the problems and formulate interventions, while the problems and solutions as perceived by those at particular risk rarely constitute the base for action."[15] Efforts to improve attendance rates should be grounded in an understanding of both supply- and demand-side barriers, elicited through engagement with affected communities.[2,16,17] The WHO Primary Health Care (PHC) Operational Framework defines engagement as "the process of involving people and communities in the design, planning and delivery of health services, thereby enabling them to make choices about care and treatment options or to participate in strategic decision-making on how health resources should be spent." Turk and colleagues note that health service interventions "must be done with, and not simply done to, the people affected."[18]

Research evidence aligns with common sense in finding that involving communities in the development of services improves health outcomes and sustainability.[18] For-profit enterprises seem to understand the value of engaging with their customers: many companies use focus groups and market research to continually hone their products and services to meet the evolving needs of their

For peer review only - http://bmjopen.bmj.com/site/about/guidelines.xhtml

customer base.[19] Our sense is that health programmes are less active in this space. Ideally - given the scale of the problem - health system managers would be able to deploy affordable, rapid, and methodologically sound tools to engage with the groups that face the highest barriers to accessing care in order to elicit their ideas for service improvements. In reality, existing qualitative elicitation and co-production techniques commonly take more than a year to plan, execute, analyse, and report.[20] They require formal ethical review, formally trained qualitative researchers, the use of specialist software, and qualitative expertise to interpret and apply the findings.[20,21] These resource requirements are prohibitive for most health system managers, and in many low-income settings there is not a ready supply of specialist expertise.[22] This can lead to well-conducted but one-off engagement activities where the findings are inappropriately generalised to other groups or -at the other end of the spectrum are tokenistic and/or methodologically flawed efforts to gather and act upon service user feedback, . We are interested in exploring whether it is possible to obtain meaningful and robust findings with rapid tools[23]; here defined as approaches that take 14 days or less “from entering the field to through to delivery of findings”[24] i.e. contacting and recruiting participants, eliciting barriers though the collection and analysis of data, and developing a list of potential interventions to improve the service, Such tools would have very wide application across a broad range of settings and would support the development of Primary Health Care (PHC) oriented systems that are built on community engagement.[25] Whilst 14 days is essentially arbitrary, it reflects an ambitious target for delivering usable intelligence that aligns with the timescales offered by market research firms to political parties and companies.[26]

Aim and objectives

We will perform a scoping review[27] of the literature to identify, categorise, and evaluate the methods that are being used to rapidly elicit barriers and potential solutions from service users in any community-based health service. We want to understand the strengths and weaknesses of the different methods that have been used, their resource requirements, and their governance frameworks as described by their users.

Responding to the need for rapid, affordable, and scientifically robust approaches that can be used to continually improve health services, we ultimately aim to identify the minimum viable product in this space. We want to identify approaches that provide sound, non-tokenistic, and actionable intelligence with minimal time, money, equipment, personnel, and skill requirements.

For peer review only - http://bmjopen.bmj.com/site/about/guidelines.xhtml

Review question

What rapid methods have been used to engage with community-based health service users to elicit barriers to access and potential solutions? For each method, what are the main outputs, methodological strengths and limitations, and resource requirements in terms of time, personnel, and other costs?For the purposes of this review, 'community-based care' will be defined as non-hospital care that involves interaction with a clinician, and a 'community' will be defined as a group who share geographies, interests, or identities. This definition is based on that used in the WHO Operational Framework for PHC and the work of Mason et al.[28] We will use the WHO Operational Framework definition for 'community engagement' that is presented in the introduction.[28] We note that Primary Health Care is not the same as community-based care: the former is a whole-of-society approach to health (that includes hospitals even though it focuses on primary care).[25]

Methods and analysis

Guidelines

Our review will be conducted in accordance with the JBI methodology, based on the principles of Arksey and O'Malley and Levac and colleagues.[29–31] Our review will be reported in line with We developed our protocol in line with the PRISMA checklist extension for scoping reviews (PRISMA-ScR, Supplementary File).[32] Scoping reviews are the most appropriate method for mapping and characterising the available evidence in a given area, and follow five steps:[33–35]

1) Defining the research question/s

2) Identifying relevant studies

3) Study selection

4) Charting the data

5) Collating, summarising, and reporting the results

An iterative approach will be taken toward searching the literature, refining the search strategy, reviewing articles for inclusion, and extracting relevant data.[32,36–38]

For peer review only - http://bmjopen.bmj.com/site/about/guidelines.xhtml

Participants

As we are concerned with barriers to access, we will focus on methods that seek to engage with those who are eligible for a given service but have not managed to attend. As such, we deem the sample population 'intended service beneficiaries' rather than 'service users'. We will include methods where engagement activities target service users and intended beneficiaries of any community-based health service in any country, serving any need. We will exclude methods that sample exclusively from attendees as – by definition – they have successfully overcome barriers to access.

We will include methods where engagement activities target lay representatives of intended service users such as patient advocacy groups, parents, or village elders, however these findings will be reported separately in the findings. We will exclude methods that exclusively engage service providers, managers, or policymakers. We will include approaches that engage a mix of users and providers as long as it is possible to disaggregate the findings pertaining to service user engagement.

As we are focusing on groups that face barriers to access, we will exclude approaches that exclusively engage with people who are present at their services i.e. our focus is on methods for contacting and engaging with non-attenders.

Concept

We are interested in methods used for engaging service users to elicit their perceptions of barriers to accessing care and generating ideas for service modifications that could improve access rates and outcomes amongst people with similar characteristics.

We are focusing on rapid methods, defined as those that can be used to deliver a list of barriers and potential solutions within 14 days or less. This is an arbitrary threshold but draws from our clinical experience in leading health services and represents what we feel to be an acceptable amount of time to generate data to inform real-time decision-making.

Given that it is not standard practice to report the length of time taken to conduct research we anticipate that our search will not identify many studies. To overcome this issue we will include studies that do not state how long they took as long as they meet all other inclusion criteria. We will analyse these studies separately.

We will include all forms of established or novel methods from any scientific field of enquiry. We expect to find examples of the following types of method:

- Interviews: face-to-face, telephone, videocall

For peer review only - http://bmjopen.bmj.com/site/about/guidelines.xhtml

- Focus groups
- Group system dynamic modelling
- Q methodology
- Nominal group technique
- Surveys: in-person, web, telephone, text message
- Rapid ethnography

Context

We are not limiting the review to any specific population, culture or geography. We will include studies from all countries and any setting except hospital inpatients. Our focus is on in-person access to existing services so we will exclude evaluations of novel services or new interventions.

We primarily define ‘access’ in terms of whether people are able to physically reach (i.e. attend) a clinical provider to get the care they need. This includes attending pre-booked appointments as well as presenting to services that do not require appointments. We will include outreach services and home-based care, but exclude virtual/digital remote consults. We will also exclude compulsory care such as when patients are sectioned for mental health care, and services where no interaction with a clinician is required, such as automated services to obtain self-testing kits.

Types of Sources

We will include all empirical study types that report on the use of a given method to elicit barriers or potential solutions with a maximum of 14 days between commencing fieldwork and generating the findings.

We will exclude methodological texts, reviews, letters, and conference abstracts. We will also exclude systematic reviews, but we will search their reference lists and include any underlying primary studies that meet our inclusion criteria.

Patient and Public Involvement

No patient and public involvement.

For peer review only - http://bmjopen.bmj.com/site/about/guidelines.xhtml

Search strategy

The search strategy will be built around rapid community-based methods and access to health services [3940](Box 1). The search will be limited to human studies published since 1978; the year of the Alma-Ata Declaration on Primary Health Care. The search will be conducted in English but we will include full-text studies published in any language. We plan to complete the review by mid-2023. The search strategy results will be presented in a PRISMA flowchart that will show how studies were eliminated until final search yield that will constitute the basis for synthesis.

We will search the following information resources: the Cochrane Library, MEDLINE Ovid, Embase Ovid and Global Health Ovid. The first 20 pages of Google Scholar will also be screened. The search strategy, including all identified keywords and index terms, will be adapted for each included database and/or information source. Box 1 presents the search strategy for Medline. The Supplementary File (Appendix) presents the tailored search strategies for all databases. We will check the reference lists of included studies and relevant systematic reviews to identify any additional potentially relevant reports of studies. Key authors will be contacted to uncover additional or upcoming studies.

Box 1: Search terms used for Medline
1. Health Services Accessibility/
2. Health Equity/
3. Social Determinants of Health/
4. (social adj2 determinant adj2 health$).tw.
5. ((health$ or social$ or racial$ or ethnic$) adj5 (inequalit$ or inequit$ or disparit$ or equit$ or disadvantage$ or depriv$)).tw.
6. (disadvant$ or marginali$ or underserved or under served or impoverish$ or minorit$ or racial$ or ethnic$).tw.
7. barrier$.tw.
8. (solution$ or improve$ or strateg$ or access$ or challeng$).ti.
9. Community-Based Participatory Research/
10. Community-Institutional Relations/
11. (communit$ adj3 (engag$ or participat$)).tw.
12. CBPR.tw.
13. (participat$ adj2 health adj2 research).tw.
14. (communit$ adj2 academic adj2 partnership$).tw.

For peer review only - http://bmjopen.bmj.com/site/about/guidelines.xhtml

15. (collective adj2 empower$).tw.
16. (equity adj2 mobili$ adj2 partnership$ adj2 communit$).tw.
17. (ethnograph$ or communitarian$).tw.
18. Interviews as Topic/
19. Patient Health Questionnaire/
20. Self Report/
21. Q-Sort/
22. Q-Sort.tw.
23. Q-methodolog$.tw.
24. (system adj2 dynamic adj2 model$).tw.
25. (nominal adj2 group$ adj2 technique$).tw.
26. or/1-25
27. Problem Solving/
28. ((rapid$ or agile) adj2 (appraisal$ or assessment$ or approach$ or evaluation$ or evaluate$ or technique$ or tool$ or method$ or research$)).tw.
29. or/27-28
30. 26 and 29
31. in vitro.tw.
32. (assay$ or microb$).tw.
33. Critical Care/
34. or/31-33
35. 30 not 34
36. limit 35 to humans
37. limit 36 to (comment or editorial or letter)
38. 36 not 37
39. limit 38 to yr="1978 -Current"

For peer review only

Study/Source of Evidence selection

Following the search, all identified citations will be collated and uploaded into Covidence (Veritas Health Innovation, Melbourne) and duplicates will be removed. Following a pilot test, titles and abstracts will then be screened by two independent reviewers (HA and RJ) for assessment against the inclusion criteria. Studies that clearly do not meet the inclusion criteria will be excluded. The reviewers will meet after every 10% batch of papers has been screened to discuss any issues. Any disagreements

For peer review only - http://bmjopen.bmj.com/site/about/guidelines.xhtml

will be resolved through consensus-based discussion, or if necessary, discussion with a third reviewer (LA).

We will obtain full texts for the potentially relevant papers. The same two review authors will independently assess the papers against the inclusion criteria to determine their eligibility for inclusion. Non-English language papers will be translated into English. The review authors will resolve disagreements through consensus-based discussion, or if necessary, discussion with the same third reviewer. The reviewers will record reasons for exclusion at the full-text screening stage.

The results of the search and the study inclusion process will be reported in full in the final scoping review and presented in a PRISMA flow diagram.[41]

Data Extraction

Two review authors (HA and RJ) will independently extract study characteristics and data from the included studies using a data extraction form developed by the reviewers. The data extraction form will be piloted on three studies by the same two review authors and required amendments will be made by consensus [42]. We anticipate a broad scope of included studies, so data charting will be an iterative process throughout the review. The data extraction tool will be modified and revised as necessary during the process of extracting data from each included evidence source. Any discrepancies will be resolved by group discussion. Modifications will be detailed in the scoping review. Where required, authors of papers will be contacted to request missing or additional data.

The data extracted will include specific details about the participants, concept, context, study methods and key findings relevant to the review question:

- Article title
- Journal title
- Authors
- Country
- Language
- Publication year
- Study type
- Type of approach (e.g. focus group) and description.
    - setting,
    - participants,
    - facilitators
- Main output if anything other than a prioritised list of potential service modifications

For peer review only - http://bmjopen.bmj.com/site/about/guidelines.xhtml

- Methodological strengths and limitations, as documented by the authors
- Resource requirements:
    - Number of personnel, and essential skills/level of training
    - Number of days for each person, full time equivalent
    - Total number of days taken from conception to findings; including planning, recruitment, engagement, and analysis stages
    - Equipment
    - Total financial cost
- Framework used to structure interaction and elicit barriers and solutions
- Method of recording (notes, audio etc)
- Other practical requirements or qualitative considerations reported in-text
- Ethics and governance requirements
- Level, form, frequency, and intensity of participation:
    - Level of participation will be assessed using the five categories used by WHO: inform, consult, involve, collaborate, and empower (Box 1).
    - Form will be assessed using the four categories used by WHO: community-oriented, -based, -managed, and –owned.
    - Frequency is defined as the number of discrete interactions between the project team and the service users.
    - Intensity represents the extent to which participants interact, exchange information and influence decision-making in participation processes [43].
- Power relations, prevailing knowledge, and beliefs and cultural barriers[18], described by the authors.
- Any documented power relations, prevailing knowledge, and beliefs and cultural barriers.

Data Analysis and Presentation

We plan to conduct a formal narrative descriptive synthesis without meta-analysis. We will stratify the synthesis by methodological approach. We will present a summary table of the different methods used, grouped by discipline. We will also tabulate the resource requirements, form of participation, and methodological strengths and limitations. Quantitative resource requirement data will be presented in whole numbers, days and 2022 US dollar amounts as appropriate. Ratios will be used to compare costs between approaches. Qualitative outcomes will be presented narratively. Methods used to engage with service users and service user representatives will be presented separately.

For peer review only - http://bmjopen.bmj.com/site/about/guidelines.xhtml

We will not conduct methodological quality assessment of included studies, in keeping with usual practice for scoping reviews.[27,29]

Limitations

Our review focuses on methods that operate extremely rapidly, using a 14 day cut-off. This choice has driven by our collective experience working with health service and system managers. We are aware that effective community engagement can often take (much) longer than 14 days, and that expediency may come at the cost of the value and nuance of the findings that are delivered. Nevertheless, just because it is unlikely that there are many, if any, robust approaches that can deliver meaningful and non-tokenistic findings within a very short timeframe, we feel it is still worth examining the literature to understand this space. There is a risk that rapid approaches produce oversimplified findings that further compound issues for marginalised groups. We will be careful to assess these risks.

Ethics and Dissemination

Ethical approval is not required. We will share our findings in the peer-reviewed literature, at conferences, and with WHO policymakers working in this space.

References

1. Dantas LF, Fleck JL, Cyrino Oliveira FL, Hamacher S. No-shows in appointment scheduling - a systematic literature review. Health Policy Amst Neth. 2018 Apr;122(4):412–21.

2. Levesque JF, Harris MF, Russell G. Patient-centred access to health care: conceptualising access at the interface of health systems and populations. Int J Equity Health. 2013 Mar 11;12(1):18.

3. Hart JT. The Inverse care law. The Lancet. 1971 Feb 27;297(7696):405–12.

4. World Health Organization. Closing the gap in a generation: health equity through action on the social determinants of health - Final report of the commission on social determinants of health [Internet]. Geneva; 2008 [cited 2021 Nov 11]. Available from: https://www.who.int/publications-detail-redirect/WHO-IER-CSDH-08.1

5. World Health Organization. Universal health coverage (UHC) [Internet]. 2021 [cited 2021 Nov 11]. Available from: https://www.who.int/news-room/fact-sheets/detail/universal-health-coverage-(uhc)

6. UN General Assembly. A/RES/70/1: Transforming our world: the 2030 Agenda for Sustainable Development [Internet]. 2015 Sep [cited 2021 Nov 11]. Available from: https://www.un.org/ga/search/view_doc.asp?symbol=A/RES/70/1&Lang=E

For peer review only - http://bmjopen.bmj.com/site/about/guidelines.xhtml

7. Frenk J. Concept and measurement of accessibility. Salud Pública México. 1992 Jan 1;27:858–64.

8. Andersen RM. Revisiting the behavioral model and access to medical care: does it matter? J Health Soc Behav. 1995 Mar;36(1):1–10.

9. Andersen R, Aday LA. Access to medical care in the U.S.: realized and potential. Med Care. 1978 Jul;16(7):533–46.

10. Penchansky R, Thomas JW. The concept of access: definition and relationship to consumer satisfaction. Med Care. 1981 Feb;19(2):127–40.

11. Cu A, Meister S, Lefebvre B, Ridde V. Assessing healthcare access using the Levesque's conceptual framework– a scoping review. Int J Equity Health. 2021 May 7;20(1):116.

12. Williams S, Barnard A, Collis P, Correia de Sousa J, Ghimire S, Habib M, et al. Remote consultations in primary care across low-, middle- and high-income countries: Implications for policy and care delivery. J Health Serv Res Policy. 2022 Dec 9;13558196221140318.

13. Paddison CA. Digital and remote primary care: the inverse care law with a 21st century twist? [Internet]. The Nuffield Trust. 2022 [cited 2023 Jan 5]. Available from: https://www.nuffieldtrust.org.uk/news-item/digital-and-remote-primary-care-the-inverse-care-law-with-a-21st-century-twist

14. Parker RF, Figures EL, Paddison CA, Matheson JI, Blane DN, Ford JA. Inequalities in general practice remote consultations: a systematic review. BJGP Open [Internet]. 2021 Jun 1 [cited 2023 Jan 5];5(3). Available from: https://bjgpopen.org/content/5/3/BJGPO.2021.0040

15. WHO Europe. Toolkit on social participation. [Internet]. 2016 [cited 2022 Apr 8]. Available from: https://www.euro.who.int/en/publications/abstracts/toolkit-on-social-participation.-methods-and-techniques-for-ensuring-the-social-participation-of-roma-populations-and-other-social-groups-in-the-design,-implementation,-monitoring-and-evaluation-of-policies-and-programmes-to-improve-their-health-2016

16. WHO and UNICEF. Declaration of Alma-Ata [Internet]. 1978 [cited 2022 Mar 23]. Available from: https://www.who.int/teams/social-determinants-of-health/declaration-of-alma-ata

17. WHO and UNICEF. Declaration of Astana on Primary Health Care [Internet]. 2018 [cited 2022 Mar 23]. Available from: https://www.who.int/teams/primary-health-care/conference/declaration

18. Turk E, Durrance-Bagale A, Han E, Bell S, Rajan S, Lota MMM, et al. International experiences with co-production and people centredness offer lessons for covid-19 responses. BMJ. 2021 Feb 15;372:m4752.

19. Statista. Market research industry [Internet]. Statista. 2022 [cited 2023 Jan 5]. Available from: https://www.statista.com/topics/1293/market-research/

20. Pope C, Mays N. Qualitative Research in Health Care, 4th Edition | Wiley [Internet]. Oxford: Wiley Blackwell; 2020 [cited 2023 Jan 5]. Available from: https://www.wiley.com/en-gb/Qualitative+Research+in+Health+Care%2C+4th+Edition-p-9781119410836

For peer review only

For peer review only - http://bmjopen.bmj.com/site/about/guidelines.xhtml

21. Creswell J, Creswell D. Research Design [Internet]. 5th ed. Thousand Oaks, Calif.: Sage; 2018 [cited 2023 Jan 5]. Available from: https://us.sagepub.com/en-us/nam/research-design/book255675

22. Franzen SRP, Chandler C, Lang T. Health research capacity development in low and middle income countries: reality or rhetoric? A systematic meta-narrative review of the qualitative literature. BMJ Open. 2017 Jan 27;7(1):e012332.

23. Miles McNall, Pennie G Foster-Fishmanhttps://journals.sagepub.com/doi/abs/10.1177/1098214007300895?journalCode=ajec https://journals.sagepub.com/doi/abs/10.1177/1098214007300895?journalCode=ajec. Methods of Rapid Evaluation, Assessment, and Appraisal. Available from: https://journals.sagepub.com/doi/abs/10.1177/1098214007300895?journalCode=ajec

24. Taylor B, Henshall C, Kenyon S, Litchfield I, Greenfield S. Can rapid approaches to qualitative analysis deliver timely, valid findings to clinical leaders? A mixed methods study comparing rapid and thematic analysis. BMJ Open. 2018 Oct 8;8(10):e019993.

25. WHO. A vision for primary health care in the 21st century: towards universal health coverage and the Sustainable Development Goals [Internet]. 2018 [cited 2022 Mar 23]. Available from: https://apps.who.int/iris/handle/10665/328065

26. Drive Research. How Quickly Can Market Research Be Completed? [Internet]. 2018 [cited 2023 Jan 5]. Available from: https://www.driveresearch.com/market-research-company-blog/how-quickly-can-market-research-be-completed/

27. Song E, Ang L, Park JY, Jun EY, Kim KH, Jun J, et al. A scoping review on biomedical journal peer review guides for reviewers. PloS One. 2021;16(5):e0251440.

28. WHO. Operational Framework for Primary Health Care Transforming Vision Into Action [Internet]. 2008. Available from: file:///C:/Users/rjonga/Downloads/9789240017832-eng.pdf

29. Peters M, Godfrey C, McInerney P. The Joanna Briggs Institute Reviewers' Manual 2015 Methodology for JBI Scoping Reviews [Internet]. Available from: https://nursing.lsuhsc.edu/JBI/docs/ReviewersManuals/Scoping-.pdf

30. Arksey H, O'Malley L. Scoping studies: towards a methodological framework. Int J Soc Res Methodol. 2005 Feb 1;8(1):19–32.

31. Levac D, Colquhoun H, O'Brien KK. Scoping studies: advancing the methodology. Implement Sci. 2010 Dec;5(1):1–9.

32. Tricco AC, Lillie E, Zarin W, O'Brien KK, Colquhoun H, Levac D, et al. PRISMA Extension for Scoping Reviews (PRISMA-ScR): Checklist and Explanation. Ann Intern Med. 2018 Oct 2;169(7):467–73.

33. Khalil H, Peters M, Godfrey CM, McInerney P, Soares CB, Parker D. An Evidence-Based Approach to Scoping Reviews. Worldviews Evid Based Nurs. 2016 Apr;13(2):118–23.

34. Munn Z, Peters MDJ, Stern C, Tufanaru C, McArthur A, Aromataris E. Systematic review or scoping review? Guidance for authors when choosing between a systematic or scoping review approach. BMC Med Res Methodol. 2018 Nov 19;18(1):143.

For peer review only - http://bmjopen.bmj.com/site/about/guidelines.xhtml

35. Peterson J, Pearce PF, Ferguson LA, Langford CA. Understanding scoping reviews: Definition, purpose, and process. J Am Assoc Nurse Pract. 2017 Jan;29(1):12–6.

36. Cooper C, Booth A, Varley-Campbell J, Britten N, Garside R. Defining the process to literature searching in systematic reviews: a literature review of guidance and supporting studies. BMC Med Res Methodol. 2018 Aug 14;18(1):85.

37. Garcia-Perdomo HA. Information sources and search strategy pitfalls to avoid in systematic reviews. Eur J Cancer Prev Off J Eur Cancer Prev Organ ECP. 2021 Nov 1;30(6):481.

38. Mueller M, D'Addario M, Egger M, Cevallos M, Dekkers O, Mugglin C, et al. Methods to systematically review and meta-analyse observational studies: a systematic scoping review of recommendations. BMC Med Res Methodol. 2018 May 21;18(1):44.

39. Said M, Boardman G, Kidd S. Barriers to accessing mental health services in Somali-Australian women: a qualitative study. Int J Ment Health Nurs. 2021 Aug;30(4):931–8.

40. Memon A, Taylor K, Mohebati LM, Sundin J, Cooper M, Scanlon T, et al. Perceived barriers to accessing mental health services among black and minority ethnic (BME) communities: a qualitative study in Southeast England. BMJ Open. 2016 Nov 16;6(11):e012337.

41. Page MJ, McKenzie JE, Bossuyt PM, Boutron I, Hoffmann TC, Mulrow CD, et al. The PRISMA 2020 statement: an updated guideline for reporting systematic reviews. BMJ. 2021 Mar 29;372:n71.

42. Cochrane Collaboration. Data Collection Form [Internet]. Available from: https://training.cochrane.org/sites/training.cochrane.org/files/public/uploads/resources/downloadable_resources/English/Collecting%20data%20-%20form%20for%20RCTs%20only.doc

43. World Health Organisation. TOOLKIT ON SOCIAL PARTICIPATION [Internet]. Available from: https://www.euro.who.int/__data/assets/pdf_file/0003/307452/Toolkit-social-partecipation.pdf

Contributions

LNA conceptualised and planned the study with SK, IG, JE, NT and AB. IG and LNA designed the search terms with input from RJ, HA, SK, JE, JR and NT. LNA wrote the first draft with JR. HA, IG, SK, JE, NT, JR and AB critically revised iterations of the manuscript. All authors read and approved the final protocol.

Funding

This work was supported by the National Institute for Health Research (NIHR) (using the UK's Official Development Assistance (ODA) Funding) and Wellcome [grant reference 215633/Z/19/Z] under the NIHR-Wellcome Partnership for Global Health Research. The views expressed are those of the authors and not necessarily those of Wellcome, the NIHR or the Department of Health and Social Care. The

For peer review only - http://bmjopen.bmj.com/site/about/guidelines.xhtml

study was sponsored by the London School of Hygiene & Tropical Medicine. The funders and study sponsor had no role in developing the protocol.

Competing interests

There are no competing interests to report for any of the authors.

For peer review only

Appendix: Search strategies

CENTRAL on the Cochrane Library

#1 MeSH descriptor: [Health Services Accessibility] this term only

#2 MeSH descriptor: [Health Equity] this term only

#3 MeSH descriptor: [Social Determinants of Health] this term only

#4 social NEAR/2 determinant NEAR/2 health*

#5 (health* or social* or racial* or ethnic*) NEAR/5 (inequalit* or inequit* or disparit* or equit* or disadvantage* or depriv*)

#6 disadvant* or marginali* or underserved or under served or impoverish* or minorit* or racial* or ethnic*

#7 barrier*

#8 (solution* or improve* or strateg* or access* or challeng*):ti

#9 MeSH descriptor: [Community-Based Participatory Research] this term only

#10 MeSH descriptor: [Community-Institutional Relations] this term only

#11 communit* NEAR/3 (engag* or participat*)

#12 CBPR

#13 participat* NEAR/2 health NEAR/2 research

#14 communit* NEAR/2 academic NEAR/2 partnership*

#15 collective NEAR/2 empower*

#16 equity NEAR/2 mobili* NEAR/2 partnership* NEAR/2 communit*

#17 ethnograph* or communitarian*

#18 MeSH descriptor: [Interviews as Topic] this term only

#19 MeSH descriptor: [Patient Health Questionnaire] this term only

#20 MeSH descriptor: [Self Report] this term only

#21 MeSH descriptor: [Q-Sort] this term only

#22 Q-Sort

#23 Q-methodolog*

#24 system NEAR/2 dynamic NEAR/2 model*

#25 nominal NEAR/2 group* NEAR/2 technique*

For peer review only

#26 #1 or #2 or #3 or #4 or #5 or #6 or #7 or #8 or #9 or #10 or #11 or #12 or #13 or #14 or #15 or #16 or #17 or #18 or #19 or #20 or #21 or #22 or #23 or #24 or #25

#27 MeSH descriptor: [Problem Solving] this term only

#28 (rapid* or agile) NEAR/2 (appraisal* or assessment* or approach* or evaluation* or evaluate* or technique* or tool* or method* or research*)

#29 #27 or #28

#30 #26 and #29

#31 in vitro

#32 assay* or microb*

#33 MeSH descriptor: [Critical Care] this term only

#34 #31 or #32 or #33

#35 #30 not #34

MEDLINE Ovid

1. Health Services Accessibility/
2. Health Equity/
3. Social Determinants of Health/
4. (social adj2 determinant adj2 health$).tw.
5. ((health$ or social$ or racial$ or ethnic$) adj5 (inequalit$ or inequit$ or disparit$ or equit$ or disadvantage$ or depriv$)).tw.
6. (disadvant$ or marginali$ or underserved or under served or impoverish$ or minorit$ or racial$ or ethnic$).tw.
7. barrier$.tw.
8. (solution$ or improve$ or strateg$ or access$ or challeng$).ti.
9. Community-Based Participatory Research/
10. Community-Institutional Relations/
11. (communit$ adj3 (engag$ or participat$)).tw.
12. CBPR.tw.
13. (participat$ adj2 health adj2 research).tw.
14. (communit$ adj2 academic adj2 partnership$).tw.
15. (collective adj2 empower$).tw.

For peer review only

16. (equity adj2 mobili$ adj2 partnership$ adj2 communit$).tw.
17. (ethnograph$ or communitarian$).tw.
18. Interviews as Topic/
19. Patient Health Questionnaire/
20. Self Report/
21. Q-Sort/
22. Q-Sort.tw.
23. Q-methodolog$.tw.
24. (system adj2 dynamic adj2 model$).tw.
25. (nominal adj2 group$ adj2 technique$).tw.
26. or/1-25
27. Problem Solving/
28. ((rapid$ or agile) adj2 (appraisal$ or assessment$ or approach$ or evaluation$ or evaluate$ or technique$ or tool$ or method$ or research$)).tw.
29. or/27-28
30. 26 and 29
31. in vitro.tw.
32. (assay$ or microb$).tw.
33. Critical Care/
34. or/31-33
35. 30 not 34
36. limit 35 to humans
37. limit 36 to (comment or editorial or letter)
38. 36 not 37

Embase Ovid

1. health care access/
2. unmet medical need/
3. health equity/
4. "social determinants of health"/

For peer review only

5. (social adj2 determinant adj2 health$).tw.
6. ((health$ or social$ or racial$ or ethnic$) adj5 (inequalit$ or inequit$ or disparit$ or equit$ or disadvantage$ or depriv$)).tw.
7. (disadvant$ or marginali$ or underserved or under served or impoverish$ or minorit$ or racial$ or ethnic$).tw.
8. barrier$.tw.
9. (solution$ or improve$ or strateg$ or access$ or challeng$).ti.
10. participatory research/
11. (communit$ adj3 (engag$ or participat$)).tw.
12. CBPR.tw.
13. (participat$ adj2 health adj2 research).tw.
14. (communit$ adj2 academic adj2 partnership$).tw.
15. (collective adj2 empower$).tw.
16. (equity adj2 mobili$ adj2 partnership$ adj2 communit$).tw.
17. (ethnograph$ or communitarian$).tw.
18. interview/
19. patient health questionnaire/
20. communication barrier/
21. Q-Sort.tw.
22. Q-methodolog$.tw.
23. (system adj2 dynamic adj2 model$).tw.
24. (nominal adj2 group$ adj2 technique$).tw.
25. or/1-24
26. problem solving/
27. ((rapid$ or agile) adj2 (appraisal$ or assessment$ or approach$ or evaluation$ or evaluate$ or technique$ or tool$ or method$ or research$)).tw.
28. or/26-27
29. 25 and 28
30. in vitro.tw.
31. (assay$ or microb$).tw.
32. intensive care/
33. or/30-32

For peer review only

34. 29 not 33
35. limit 34 to human
36. limit 35 to (conference abstract or conference paper or editorial or letter)
37. 35 not 36

Global Health Ovid

1. health determinants/
2. empowerment/
3. (social adj2 determinant adj2 health$).tw.
4. ((health$ or social$ or racial$ or ethnic$) adj5 (inequalit$ or inequit$ or disparit$ or equit$ or disadvantage$ or depriv$)).tw.
5. (disadvant$ or marginali$ or underserved or under served or impoverish$).tw.
6. barrier$.tw.
7. (solution$ or improve$ or strateg$ or access$ or challeng$).ti.
8. (communit$ adj3 (engag$ or participat$)).tw.
9. CBPR.tw.
10. (participat$ adj2 health adj2 research).tw.
11. (communit$ adj2 based adj2 research).tw.
12. (communit$ adj2 academic adj2 partnership$).tw.
13. (collective adj2 empower$).tw.
14. (equity adj2 mobili$ adj2 partnership$ adj2 communit$).tw.
15. (ethnograph$ or communitarian$).tw.
16. interviews/
17. questionnaires/
18. Q-Sort.tw.
19. Q-methodolog$.tw.
20. (system adj2 dynamic adj2 model$).tw.
21. (nominal adj2 group$ adj2 technique$).tw.
22. or/1-21

For peer review only

23. Problem Solving/
24. ((rapid$ or agile) adj2 (appraisal$ or assessment$ or approach$ or evaluation$ or evaluate$ or technique$ or tool$ or method$ or research$)).tw.
25. or/23-24
26. 22 and 25
27. man.od.
28. 26 and 27
29. in vitro.tw.
30. (assay* or microb*).tw.
31. intensive care/ or intensive care units/
32. animals.od.
33. plants.od.
34. or/29-33
35. 28 not 34
36. limit 35 to (abstract only or conference or conference paper or conference proceedings or editorial)
37. 35 not 36

For peer review only

Appendix: Search strategies

CENTRAL on the Cochrane Library

#1 MeSH descriptor: [Health Services Accessibility] this term only

#2 MeSH descriptor: [Health Equity] this term only

#3 MeSH descriptor: [Social Determinants of Health] this term only

#4 social NEAR/2 determinant NEAR/2 health*

#5 (health* or social* or racial* or ethnic*) NEAR/5 (inequalit* or inequit* or disparit* or equit* or disadvantage* or depriv*)

#6 disadvant* or marginali* or underserved or under served or impoverish* or minorit* or racial* or ethnic*

#7 barrier*

#8 (solution* or improve* or strateg* or access* or challeng*):ti

#9 MeSH descriptor: [Community-Based Participatory Research] this term only

#10 MeSH descriptor: [Community-Institutional Relations] this term only

#11 communit* NEAR/3 (engag* or participat*)

#12 CBPR

#13 participat* NEAR/2 health NEAR/2 research

#14 communit* NEAR/2 academic NEAR/2 partnership*

#15 collective NEAR/2 empower*

#16 equity NEAR/2 mobili* NEAR/2 partnership* NEAR/2 communit*

#17 ethnograph* or communitarian*

#18 MeSH descriptor: [Interviews as Topic] this term only

#19 MeSH descriptor: [Patient Health Questionnaire] this term only

#20 MeSH descriptor: [Self Report] this term only

#21 MeSH descriptor: [Q-Sort] this term only

#22 Q-Sort

#23 Q-methodolog*

#24 system NEAR/2 dynamic NEAR/2 model*

#25 nominal NEAR/2 group* NEAR/2 technique*

For peer review only

#26 #1 or #2 or #3 or #4 or #5 or #6 or #7 or #8 or #9 or #10 or #11 or #12 or #13 or #14 or #15 or #16 or #17 or #18 or #19 or #20 or #21 or #22 or #23 or #24 or #25

#27 MeSH descriptor: [Problem Solving] this term only

#28 (rapid* or agile) NEAR/2 (appraisal* or assessment* or approach* or evaluation* or evaluate* or technique* or tool* or method* or research*)

#29 #27 or #28

#30 #26 and #29

#31 in vitro

#32 assay* or microb*

#33 MeSH descriptor: [Critical Care] this term only

#34 #31 or #32 or #33

#35 #30 not #34

MEDLINE Ovid

1. Health Services Accessibility/
2. Health Equity/
3. Social Determinants of Health/
4. (social adj2 determinant adj2 health$).tw.
5. ((health$ or social$ or racial$ or ethnic$) adj5 (inequalit$ or inequit$ or disparit$ or equit$ or disadvantage$ or depriv$)).tw.
6. (disadvant$ or marginali$ or underserved or under served or impoverish$ or minorit$ or racial$ or ethnic$).tw.
7. barrier$.tw.
8. (solution$ or improve$ or strateg$ or access$ or challeng$).ti.
9. Community-Based Participatory Research/
10. Community-Institutional Relations/
11. (communit$ adj3 (engag$ or participat$)).tw.
12. CBPR.tw.
13. (participat$ adj2 health adj2 research).tw.
14. (communit$ adj2 academic adj2 partnership$).tw.
15. (collective adj2 empower$).tw.

For peer review only

16. (equity adj2 mobili$ adj2 partnership$ adj2 communit$).tw.
17. (ethnograph$ or communitarian$).tw.
18. Interviews as Topic/
19. Patient Health Questionnaire/
20. Self Report/
21. Q-Sort/
22. Q-Sort.tw.
23. Q-methodolog$.tw.
24. (system adj2 dynamic adj2 model$).tw.
25. (nominal adj2 group$ adj2 technique$).tw.
26. or/1-25
27. Problem Solving/
28. ((rapid$ or agile) adj2 (appraisal$ or assessment$ or approach$ or evaluation$ or evaluate$ or technique$ or tool$ or method$ or research$)).tw.
29. or/27-28
30. 26 and 29
31. in vitro.tw.
32. (assay$ or microb$).tw.
33. Critical Care/
34. or/31-33
35. 30 not 34
36. limit 35 to humans
37. limit 36 to (comment or editorial or letter)
38. 36 not 37

Embase Ovid

1. health care access/
2. unmet medical need/
3. health equity/
4. "social determinants of health"/

For peer review only

5. (social adj2 determinant adj2 health$).tw.
6. ((health$ or social$ or racial$ or ethnic$) adj5 (inequalit$ or inequit$ or disparit$ or equit$ or disadvantage$ or depriv$)).tw.
7. (disadvant$ or marginali$ or underserved or under served or impoverish$ or minorit$ or racial$ or ethnic$).tw.
8. barrier$.tw.
9. (solution$ or improve$ or strateg$ or access$ or challeng$).ti.
10. participatory research/
11. (communit$ adj3 (engag$ or participat$)).tw.
12. CBPR.tw.
13. (participat$ adj2 health adj2 research).tw.
14. (communit$ adj2 academic adj2 partnership$).tw.
15. (collective adj2 empower$).tw.
16. (equity adj2 mobili$ adj2 partnership$ adj2 communit$).tw.
17. (ethnograph$ or communitarian$).tw.
18. interview/
19. patient health questionnaire/
20. communication barrier/
21. Q-Sort.tw.
22. Q-methodolog$.tw.
23. (system adj2 dynamic adj2 model$).tw.
24. (nominal adj2 group$ adj2 technique$).tw.
25. or/1-24
26. problem solving/
27. ((rapid$ or agile) adj2 (appraisal$ or assessment$ or approach$ or evaluation$ or evaluate$ or technique$ or tool$ or method$ or research$)).tw.
28. or/26-27
29. 25 and 28
30. in vitro.tw.
31. (assay$ or microb$).tw.
32. intensive care/
33. or/30-32

For peer review only

34. 29 not 33
35. limit 34 to human
36. limit 35 to (conference abstract or conference paper or editorial or letter)
37. 35 not 36

Global Health Ovid

1. health determinants/
2. empowerment/
3. (social adj2 determinant adj2 health$).tw.
4. ((health$ or social$ or racial$ or ethnic$) adj5 (inequalit$ or inequit$ or disparit$ or equit$ or disadvantage$ or depriv$)).tw.
5. (disadvant$ or marginali$ or underserved or under served or impoverish$).tw.
6. barrier$.tw.
7. (solution$ or improve$ or strateg$ or access$ or challeng$).ti.
8. (communit$ adj3 (engag$ or participat$)).tw.
9. CBPR.tw.
10. (participat$ adj2 health adj2 research).tw.
11. (communit$ adj2 based adj2 research).tw.
12. (communit$ adj2 academic adj2 partnership$).tw.
13. (collective adj2 empower$).tw.
14. (equity adj2 mobili$ adj2 partnership$ adj2 communit$).tw.
15. (ethnograph$ or communitarian$).tw.
16. interviews/
17. questionnaires/
18. Q-Sort.tw.
19. Q-methodolog$.tw.
20. (system adj2 dynamic adj2 model$).tw.
21. (nominal adj2 group$ adj2 technique$).tw.
22. or/1-21

For peer review only

23. Problem Solving/
24. ((rapid$ or agile) adj2 (appraisal$ or assessment$ or approach$ or evaluation$ or evaluate$ or technique$ or tool$ or method$ or research$)).tw.
25. or/23-24
26. 22 and 25
27. man.od.
28. 26 and 27
29. in vitro.tw.
30. (assay* or microb*).tw.
31. intensive care/ or intensive care units/
32. animals.od.
33. plants.od.
34. or/29-33
35. 28 not 34
36. limit 35 to (abstract only or conference or conference paper or conference proceedings or editorial)
37. 35 not 36

For peer review only

Preferred Reporting Items for Systematic reviews and Meta-Analyses extension for Scoping Reviews (PRISMA-ScR) Checklist

SECTION	ITEM	PRISMA-ScR CHECKLIST ITEM	REPORTED ON PAGE #
**TITLE**			
Title	1	Identify the report as a scoping review.	Page 1
**ABSTRACT**			
Structured summary	2	Provide a structured summary that includes (as applicable): background, objectives, eligibility criteria, sources of evidence, charting methods, results, and conclusions that relate to the review questions and objectives.	Page 3
**INTRODUCTION**			
Rationale	3	Describe the rationale for the review in the context of what is already known. Explain why the review questions/objectives lend themselves to a scoping review approach.	Page 4-5
Objectives	4	Provide an explicit statement of the questions and objectives being addressed with reference to their key elements (e.g., population or participants, concepts, and context) or other relevant key elements used to conceptualize the review questions and/or objectives.	4-6
**METHODS**			
Protocol and registration	5	Indicate whether a review protocol exists; state if and where it can be accessed (e.g., a Web address); and if available, provide registration information, including the registration number.	N/A
Eligibility criteria	6	Specify characteristics of the sources of evidence used as eligibility criteria (e.g., years considered, language, and publication status), and provide a rationale.	Page 7-8
Information sources*	7	Describe all information sources in the search (e.g., databases with dates of coverage and contact with authors to identify additional sources), as well as the date the most recent search was executed.	Page 8-10
Search	8	Present the full electronic search strategy for at least 1 database, including any limits used, such that it could be repeated.	Page 10
Selection of sources of evidence†	9	State the process for selecting sources of evidence (i.e., screening and eligibility) included in the scoping review.	Page 11
Data charting process‡	10	Describe the methods of charting data from the included sources of evidence (e.g., calibrated forms or forms that have been tested by the team before their use, and whether data charting was done independently or in duplicate) and any processes for obtaining and confirming data from investigators.	Page 11-12
Data items	11	List and define all variables for which data were sought and any assumptions and simplifications made.	Page 11, 18-37
Critical appraisal of individual sources of evidence§	12	If done, provide a rationale for conducting a critical appraisal of included sources of evidence; describe the methods used and how this information was used in any data synthesis (if appropriate).	Page 18-37

SECTION	ITEM	PRISMA-ScR CHECKLIST ITEM	REPORTED ON PAGE #
Synthesis of results	13	Describe the methods of handling and summarizing the data that were charted.	Page 13
**RESULTS**			
Selection of sources of evidence	14	Give numbers of sources of evidence screened, assessed for eligibility, and included in the review, with reasons for exclusions at each stage, ideally using a flow diagram.	Page 7-9
Characteristics of sources of evidence	15	For each source of evidence, present characteristics for which data were charted and provide the citations.	Page 18-37
Critical appraisal within sources of evidence	16	If done, present data on critical appraisal of included sources of evidence (see item 12).	Page 18-37
Results of individual sources of evidence	17	For each included source of evidence, present the relevant data that were charted that relate to the review questions and objectives.	Page 18-37, 38-45
Synthesis of results	18	Summarize and/or present the charting results as they relate to the review questions and objectives.	Page 38-40
**DISCUSSION**			
Summary of evidence	19	Summarize the main results (including an overview of concepts, themes, and types of evidence available), link to the review questions and objectives, and consider the relevance to key groups.	Page 38-58
Limitations	20	Discuss the limitations of the scoping review process.	Page 59
Conclusions	21	Provide a general interpretation of the results with respect to the review questions and objectives, as well as potential implications and/or next steps.	Page 60-61
**FUNDING**			
Funding	22	Describe sources of funding for the included sources of evidence, as well as sources of funding for the scoping review. Describe the role of the funders of the scoping review.	Page 2

JBI = Joanna Briggs Institute; PRISMA-ScR = Preferred Reporting Items for Systematic reviews and Meta-Analyses extension for Scoping Reviews.

* Where *sources of evidence* (see second footnote) are compiled from, such as bibliographic databases, social media platforms, and Web sites.

† A more inclusive/heterogeneous term used to account for the different types of evidence or data sources (e.g., quantitative and/or qualitative research, expert opinion, and policy documents) that may be eligible in a scoping review as opposed to only studies. This is not to be confused with *information sources* (see first footnote).

‡ The frameworks by Arksey and O'Malley (6) and Levac and colleagues (7) and the JBI guidance (4, 5) refer to the process of data extraction in a scoping review as data charting.

§ The process of systematically examining research evidence to assess its validity, results, and relevance before using it to inform a decision. This term is used for items 12 and 19 instead of "risk of bias" (which is more applicable to systematic reviews of interventions) to include and acknowledge the various sources of evidence that may be used in a scoping review (e.g., quantitative and/or qualitative research, expert opinion, and policy document).

From: Tricco AC, Lillie E, Zarin W, O'Brien KK, Colquhoun H, Levac D, et al. PRISMA Extension for Scoping Reviews (PRISMAScR): Checklist and Explanation. Ann Intern Med. 2018;169:467–473. doi: 10.7326/M18-0850.

St. Michael's
Inspired Care.
Inspiring Science.

For peer review only - http://bmjopen.bmj.com/site/about/guidelines.xhtml
